# Gradually Increased Training Intensity Benefits Rehabilitation Outcome after Stroke by BDNF Upregulation and Stress Suppression

**DOI:** 10.1155/2014/925762

**Published:** 2014-06-19

**Authors:** Jing Sun, Zheng Ke, Shea Ping Yip, Xiao-ling Hu, Xiao-xiang Zheng, Kai-yu Tong

**Affiliations:** ^1^Interdisciplinary Division of Biomedical Engineering, The Hong Kong Polytechnic University, Hong Kong; ^2^Department of Health Technology and Informatics, The Hong Kong Polytechnic University, Hong Kong; ^3^College of Biomedical Engineering and Instrument Science, Zhejiang University, Hangzhou 310027, China

## Abstract

Physical training is necessary for effective rehabilitation in the early poststroke period. Animal studies commonly use fixed training intensity throughout rehabilitation and without adapting it to the animals' recovered motor ability. This study investigated the correlation between training intensity and rehabilitation efficacy by using a focal ischemic stroke rat model. Eighty male Sprague-Dawley rats were induced with middle cerebral artery occlusion/reperfusion surgery. Sixty rats with successful stroke were then randomly assigned into four groups: control (CG, *n* = 15), low intensity (LG, *n* = 15), gradually increased intensity (GIG, *n* = 15), and high intensity (HG, *n* = 15). Behavioral tests were conducted daily to evaluate motor function recovery. Stress level and neural recovery were evaluated via plasma corticosterone and brain-derived neurotrophic factor (BDNF) concentration, respectively. GIG rats significantly (*P* < 0.05) recovered motor function and produced higher hippocampal BDNF (112.87 ± 25.18 ng/g). GIG and LG rats exhibited similar stress levels (540.63 ± 117.40 nM/L and 508.07 ± 161.30 nM/L, resp.), which were significantly lower (*P* < 0.05) than that (716.90 ± 156.48 nM/L) of HG rats. Training with gradually increased intensity achieved better recovery with lower stress. Our observations indicate that a training protocol that includes gradually increasing training intensity should be considered in both animal and clinical studies for better stroke recovery.

## 1. Introduction

Stroke is the third cause of death after cancer and cardiac diseases [1] and is the leading cause of adult disability in many countries [2]. Ischemic stroke accounts for more than 80% of episodes among patients [1]. Hemiparesis is an inconvenient symptom common in stroke survivors. According to the Hong Kong Authority Statistical Report 2009-2010, the number of annual stroke admissions to public hospitals increased from 24,743 cases in 2005 to 25,614 cases in 2009 [3]. A growing elderly population vulnerable to stroke [2, 4] substantially increases medical care burden in Hong Kong and the developed countries. Thus, effective rehabilitation is essential to help stroke survivors regain impaired motor function for improved quality of life.

Poststroke functional motor training, with repetitive attempts to move paretic limbs, assists the stroke-damaged efferent pathways more effectively in the subacute stroke period when the brain network is sensitive to therapeutic interventions [5]. Studies on efficacy, mechanism, and comparisons of training methods have been performed for many years [6–8]. Training after stroke benefits motor function recovery and promotes neurorehabilitation [8, 9]. Treadmill training, a conventional and easy method, has been employed in both human trials and animal models [10, 11]. Poststroke treadmill training is continually used in rehabilitation due to its effectiveness in both functional mobility and cardiovascular fitness in patients with chronic stroke [11]. Early treadmill training could also reduce brain infarct volume and improve neurologic function compared to spontaneous recovery in stroke rat models [12, 13]. Moderate treadmill training could upregulate brain-derived neurotrophic factor (BDNF) [14].

BDNF is a protein discovered in the early 1980s which is encoded by BDNF gene and expressed broadly in the central and the peripheral nervous systems [15]. BDNF is one member of the “neurotrophin” family of growth factors that is believed to support the neuron survival and encourages growth and differentiation of new neurons and synapses [16]. BDNF is active in the hippocampus, a region vital to learning, memory, and higher thinking [17]. BDNF is related to neuroplasticity contributing to motor learning, recovery, and neural rehabilitation after stroke [5]. Stroke induces the loss of motor function, and rehabilitation is the process of relearning; thus, higher BDNF concentration in the brain implies learning and neural rehabilitation [18].

Treadmill training can cause stress, leading to a series of physical changes that inhibit neural recovery during rehabilitation [19, 20]. Animals suffer from stress and actually develop similar pathology to humans [21]. Plasma corticosterone (CORT) concentration is widely used as a biomarker of stress in animal models [7, 22, 23]. CORT could downregulate BDNF level in hippocampus [24]. Due to the controversial function of treadmill training after stroke, it is important to evaluate its effect on early stroke physical rehabilitation and the relationship between training loads, motor recovery, and stress levels. We, therefore, designed this study to investigate these relationships via an ischemic stroke rat model.

Intensity is thought to be a key factor in treadmill training and is associated with stress. High-speed treadmill training induces high CORT levels in a stroke rat model [18]. Stress endurance is also enhanced by exercise [25]. Thus, stress level may not only depend on training intensity but also be influenced by subjects' conditions. Moreover, adjusted training intensity may be directly correlated to rehabilitation outcomes.

Effectiveness of treadmill training intensity in motor function recovery and neurorehabilitation has not yet been completely elucidated. Both clinical and animal studies have focused on fixed training intensity [26, 27]. It remains unclear whether varied training intensity is more effective. In this study, we employed a focal ischemic stroke rat model to evaluate the effect of differing treadmill training intensities on motor function recovery and neurorehabilitation. We also analyzed CORT and BDNF levels in early stroke phase. A gradually increased training intensity was designed to investigate the relationship between intensity, motor recovery, and stress level. This study extends our understanding of treadmill training intensity and influences rehabilitation program design.

## 2. Methodology

Eighty male Sprague-Dawley (SD) rats (between 2 and 3 months) weighing 280–360 g were used in this study. Rats had free access to food and drink throughout the experimental period. All procedures were approved by the “Animals Subject Ethics Sub-Committee” of the Hong Kong Polytechnic University and conformed to the guidelines on the care and ethical use of experimental animals [28].

**Figure 1:**
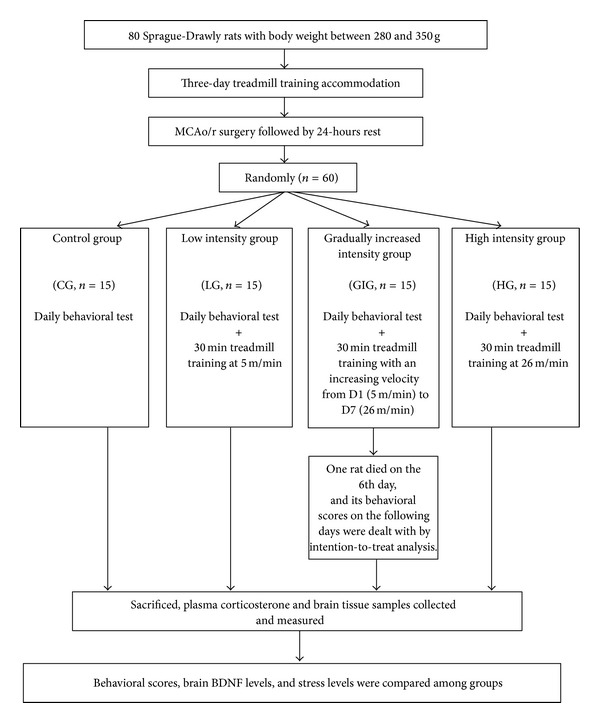
Flowchart of the experimental design.

**Figure 2:**
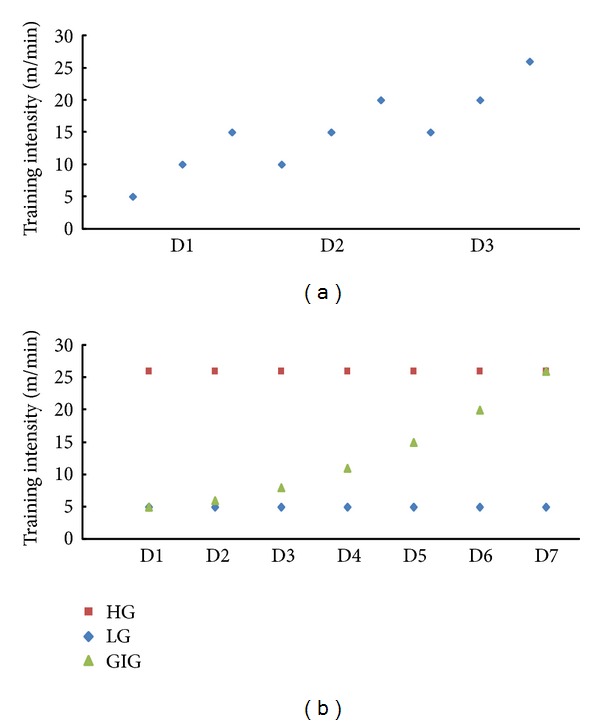
Training intensity setup for (a) the 3-day accommodation and (b) poststroke training.

Rats were trained for three days (Figure 1; accommodation protocol in Figure 2(a)) to become accustomed to treadmill exercises. Rats unable to run on the treadmill were removed from the experiment. After three days, rats underwent middle cerebral artery occlusion/reperfusion surgery (MCAo/r) to induce ischemic stroke. After 24 hours, successfully induced stroke rats (*n* = 60) with motor impairment using Longa's test [29] and behavioral core between 1 and 3 were randomly assigned into 4 groups: control (CG, *n* = 15), low training intensity (LG, *n* = 15), gradually increasing training intensity (GIG, *n* = 15), and high training intensity (HG, *n* = 15). Rats in CG were fed in standard cages for one week, while the rest underwent daily treadmill training with different training intensities. LG rats were allowed to run on the treadmill for 30 minutes with a 10-minute rest between 10 minutes of running section at a velocity of 5 m/min. HG rats ran at 26 m/min with the same training and rest regimens. Rats in GIG ran from 5 m/min on the 1st day (D1) up to 26 m/min on the last day (D7). Daily behavioral scores were recorded via a skilled researcher blind to group assignment. On the last intervention day, rats were anesthetized and sacrificed via decapitation within two hours after the last training. Trunk blood and brain tissues from the hippocampus, striatum, and sensorimotor cortex were collected. Trunk blood samples were immediately centrifuged to acquire plasma. Brain tissue samples were processed according to a standard BDNF sample preparation protocol (Promega, USA). Plasma and brain tissue samples were used for CORT and BDNF detection, respectively.

### 2.1. Middle Cerebral Artery Occlusion/Reperfusion (MCAo/r) Surgery

The MCAo/r surgery induced focal ischemic stroke rat model by Koizumi [30] in 1986 was employed in this study. Surgery mimicked practices by Ke et al. [7]. Briefly, rats in all groups were anesthetized with 10% chloral hydrate (0.4 mg/kg for induction and 0.02 mg subsequently). Incisions were made at the neck midline to expose the common carotid artery (CCA), and then the external carotid artery was ligated. Subsequently, a commercial filament with a tip diameter of 0.39 ± 0.02 mm (Beijing Sunbio Biotech, China) was inserted into the CCA and advanced along the internal carotid artery until the tip of the filament reached the middle cerebral artery. Occlusion lasted for 60 minutes after which the filament was then withdrawn to allow reperfusion. Six hours after MCAo/r surgery, rats were examined for neurological deficit level using Longa's test. For Longa's test, a score of 0 indicates no stroke and 4 represents severe stroke [29]. Rats with a score between 1 and 3 were enrolled in the experiment and kept in individual cages.

### 2.2. Treadmill Training Intervention

Treadmill training intensity was suggested to affect memory function recovery which is related to neural activity in the hippocampus [31]. Different training intensities bring different stress levels to rats [7]. Velocity is a determining factor in intensity and workload. Different velocities generated different training intensities. In previous studies, training intensities mainly were set from 2 m/min to 30 m/min and the daily training time length was mainly set to 30 minutes [6, 8, 31]. In this study, total training time was fixed at 30 minutes, and 5 m/min and 26 m/min were chosen as low and high treadmill training velocities, respectively. In LG and HG, rats ran at a constant velocity through 7 days of training at 5 m/min and 26 m/min, respectively. Rats are generally weak the first several days after stroke but can spontaneously recover [7], gaining better motor function with time. Therefore, the study designated a rat group to gradually increasing intensity from low speed (5 m/min) on the first day to high speed (26 m/min) on the seventh day. Velocity increased slowly in GIG the first four days, and for the following three days, it increased relatively faster. The training setup for all groups is shown in Figure 2(b).

### 2.3. Motor Function Test

Motor function improvement was evaluated by the De Ryck behavioral test [32] on daily basis throughout the 7-day intervention. Six out of eight tasks evaluate functions including postural reflex, visual placing in the forward and sideways directions, tactile placing of the dorsal and lateral paw surfaces, and proprioceptive placing; the other two tasks examine hindlimb's tactile placing of lateral paw surfaces and proprioceptive placing. The score for each subtask ranges from 0 to 2 with the higher score indicating better motor function. Compared to normal rats, stroke rats cannot place injured limbs normally. Thus, placement function of injured forelimbs and hindlimbs was assessed through the tasks by a skilled researcher blind to group assignments.

### 2.4. Brain BDNF and Plasma Corticosterone Detection

Brain BDNF and plasma CORT were evaluated using an enzyme-linked immunosorbent assay (ELISA) [6, 7, 33, 34]. All rats were anesthetized within two hours after the last intervention and sacrificed via decapitation. Trunk blood was collected and centrifuged to obtain plasma. The brain was carefully extracted from the skull, and brain tissues including hippocampus, striatum, and affected sensorimotor cortex were then obtained. BDNF Emax ImmunoAssay System (Promega, USA) was used to measure BDNF concentrations. Plasma CORT concentrations were quantified via Cayman's CORT EIA Kit (Cayman, USA).

### 2.5. Statistical Analysis

All results were expressed as means ± standard deviations. SPSS (IBM, version 20) was used for data analysis and the level of statistical significance was set at P = 0.05. Intention-to-treat analysis was used for any rat that died during the intervention period. The Shapiro-Wilk test was used to examine the normality of all results. Two-way repeated measures analysis of variance (ANOVA) with baseline as covariate and the Bonferroni post hoc test were used to compare motor function scores. One-way ANOVA test was used to compare CORT and BDNF concentrations.

## 3. Results

Sixty rats underwent successful MCAo/r surgery that induced motor impairment within 24 hours. Stroke rats were randomly assigned into 4 groups (CG, LG, HG, and GIG) with 15 rats in each group. Throughout the experiment, only one rat in the GIG group died on the 6th day. Its behavioral scores on the last two days were the same as that on the 5th day based on the intention-to-treat principle. Results of behavioral scores, CORT concentrations, and BNDF concentrations are shown in Table 1. Through the Shapiro-Wilk test, all results including behavioral scores, CORT concentrations, and BDNF concentrations showed normal distributions (*P* > 0.05). All results were then used for further analysis.

### 3.1. Motor Function Recovery

**Figure 3:**
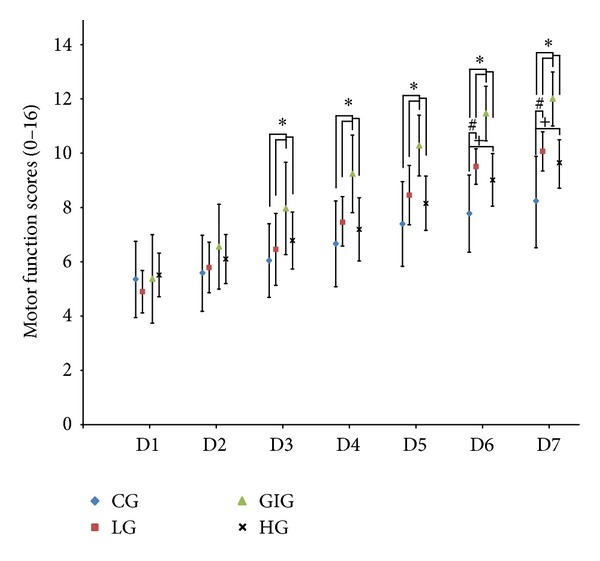
Behavioral scores over the seven-day intervention. A higher behavioral score indicates better motor function. *: significant difference observed via 2-way repeated measures ANOVA with the score on D1 as covariate when comparing GIG to the other three groups. ^#, +^: significant difference observed via 2-way repeated measures ANOVA with the score on D1 as covariate when comparing LG and HG to CG, respectively. From D3 to D7, rats in GIG scored significantly higher for behavioral score compared to LG, HG, and CG, while rats in LG and HG showed better motor function on D6 and D7 compared to CG. Results suggested that gradually increased training intensity can facilitate motor function recovery during the subacute stroke period.

Behavioral scores indicating motor function recovery over the experimental period are presented in Figure 3. Significant differences existed among the four groups. GIG rats showed significantly higher behavioral scores from the 3rd to the last day compared to those in the other groups. Rats in LG and HG also exhibited significantly better motor function recovery from the 6th day than the control.

### 3.2. Brain BDNF and Plasma Corticosterone Concentrations

**Figure 4:**
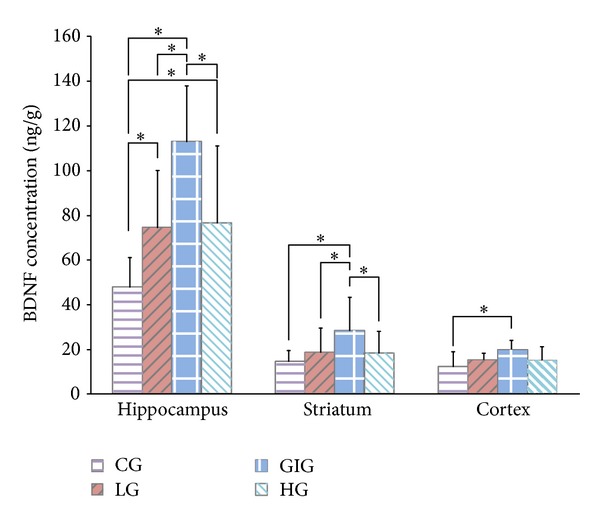
BDNF concentration in the hippocampus, striatum, and cortex. *: significant differences between groups acquired via one-way ANOVA with post hoc test. The hippocampus showed significantly higher BDNF level than striatum and cortex in all groups. GIG rats exhibited significantly higher BDNF levels in the hippocampus and striatum than the other groups. Significantly different cortical BDNF levels were only observed between the GIG and the CG. BDNF levels in LG and HG rats were not apparently different in all of the brain tissues; both were significantly higher than CG in the hippocampus and striatum.

**Figure 5:**
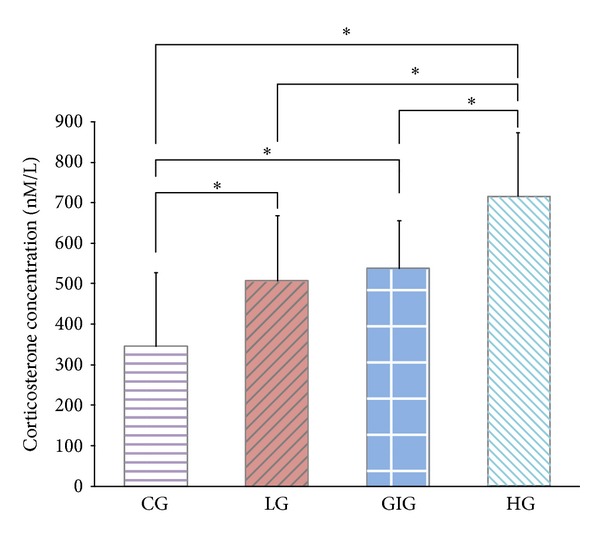
Plasma corticosterone concentrations on the last day. Higher values represent higher stress. *: significant differences between groups acquired via one-way ANOVA with post hoc test. HG rats were stressed the most, and LG and GIG rats showed similar stress levels which were significantly higher than CG. Although GIG rats ran at the same velocity as HG, their average stress level was significantly lower.

Hippocampal BDNF concentrations were significantly higher than in both the striatum and cortex for all groups. GIG rats showed the highest BDNF levels in the hippocampus and striatum in Figure 4. Significantly different cortical BDNF levels were observed between GIG and CG rats. BDNF levels in LG and HG rats were not apparently different but were significantly higher in the hippocampus and striatum than CG rats (Figure 4).  Figure 5 shows plasma CORT concentrations. Rats in the 3 training groups exhibited significantly higher CORT levels over control. CORT levels in GIG rats were significantly lower than HG but similar to LG.

## 4. Discussion

We show that treadmill training intensities for ischemic stroke rats affect motor function recovery, BDNF concentration, and stress level over the 7-day intervention. We set up three training intensity levels including low, high, and gradually increased intensity from low to high. Gradually increased training intensity (GIG) induced significantly better motor function recovery. Rats in this group showed similar stress levels in comparison to LG, but BDNF concentrations in brain tissues (hippocampus and striatum) were significantly higher than LG. Rats in HG were stressed more than LG; however, functional recovery rates were similar to LG and significantly lower than GIG. Results indicated that rats with treadmill gradually increased intensities better regain motor function recovery.

**Figure 6:**
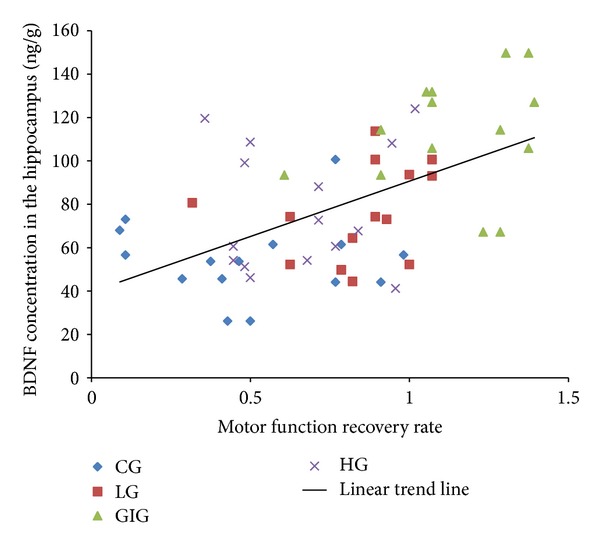
Correlation between hippocampal BDNF concentration and motor function recovery rate. BDNF levels in the hippocampus correlated with motor function recovery rate, meaning that BDNF levels could indicate motor function recovery rate.

Consistent with other studies, BDNF levels were lower in striatum and cortex than in the hippocampus [7, 35]. The hippocampus plays an important role in learning and memory, and rehabilitation is a process of relearning, making hippocampal neurons active [17]. BDNF level is highly related to neural survival, growth, and differentiation [16], probably producing a high hippocampal BDNF level. GIG rats showed the highest BDNF concentrations in the hippocampus and striatum and had the best motor function recovery. Importantly, we found a significantly positive relationship (correlation coefficient: 0.537; *P* < 0.01) between motor function recovery rate and hippocampal BDNF concentrations (Figure 6). BDNF has been used to treat photothrombotic stroke rats and it improved motor function recovery when compared to spontaneous recovery [36]. Other studies also show that higher BDNF level in the brain indicates better motor function recovery after stroke [7, 37]. Our results remain consistent with those of previous studies. Significant higher BDNF levels were found in GIG rats, leading to significantly better motor function recovery. Similar BDNF levels were observed in LG and HG rats that showed similar motor function recovery.

Ploughman considered exercise brain food that ultimately enhances brain functions like memory and learning [38]. Additionally, Ploughman et al. [39] suggest that moderate exercise has positive effects on physically disabled young people aided by their high brain plasticity. Both prolonged and short-term moderate exercises increase hippocampal BDNF levels and brain mitochondrial biogenesis in rats [14, 40, 41]. Physical training for stroke rat models was reported to facilitate motor function recovery and upregulate BDNF levels [42]. Four-week consecutive low-speed treadmill training started on the 4th day after stroke was found to improve hippocampal function in a MCAo induced stroke rat model [31]. Thus, exercise seems to upregulate brain BDNF concentrations, a result supported by this study. Moreover, GIG training improves BDNF production in brain tissues after stroke, indicating better brain function recovery.

Early physical training facilitates rehabilitation after stroke, but it is also a source of stress that mediates BDNF regulation. CORT is a steroid hormone produced by the hypothalamic-pituitary-adrenal axis and is released into the blood. Adrenalectomized Wistar rats were used to investigate the time course and dose-dependency of CORT's effect on BDNF mRNA and protein, with results showing short-term corticosterone concentration changes having transient and dose-dependent downregulation effects for both hippocampal BDNF mRNA and protein [24]. Forced treadmill training induces stress and has been suggested to lower physical rehabilitation and BDNF levels in the hippocampus compared to voluntary wheel running; yet it still stimulates functional recovery [7]. Treadmill training intensity could affect memory function recovery, while the hippocampus determines memory function [31]. Training intensity, thus, may affect hippocampal activity. Stress level is highly related to training intensity. High training intensity causes significantly high stress level, as a result of our study. Hippocampal BDNF level could represent neural activity in the hippocampus. Higher BDNF levels in the hippocampus indicate better neural activity. Thus, stress level may correlate with hippocampal BNDF level. On one hand, in this study, consistent low and high training intensity induced low and high stress levels associated with similar hippocampal BDNF level; however, gradually increased intensity induced stress levels between low and high intensities and close to low intensity: stress may inhibit brain BDNF production. On the other hand, rats with low training intensity were stressed significantly more than those without training but still exhibited significantly better motor function recovery, suggesting that stress was not the only factor mediating BDNF production during rehabilitation. Exercise should be another important factor determining rehabilitation outcomes. It could increase muscle and brain mitochondrial biogenesis, strengthening fatigue resistance and endurance performance [25]. In this study, GIG training may better improve stress endurance and it obtained better recovery. Training intensity, thus, should be appropriately chosen for better recovery after stroke.

Repeated training is an important tool applied widely in clinics and laboratories to improve recovery after stroke. Intensity in forced training is a critical stress-inducing factor. We thus designed a gradually increasing treadmill training intensity regimen for stroke rats. We found that the training intensity should be designed to match recovery rate and minimize stress. Training with gradually increased intensity can produce significantly better motor function rehabilitation compared to stably low and high training intensity. We extended the understanding of the importance of training intensity in rehabilitation after stroke. A training protocol that includes gradually increasing training intensity should be considered in both animal and clinical human studies.

## Figures and Tables

**Table 1:** An Overview of Rehabilitation Outcomes of Motor Function, BDNF Levels in Hippocampus, Striatum and Cortex, and Plasma Corticosterone (CORT).

Items	Group	Pre-training	Post-training	Post hoc (*P*)
Motor Function				CG vs LG (0.018∗)
	CG	5.36 ± 1.41	8.23 ± 1.69	CG vs GIG (<0.001∗)
	LG	4.89 ± 0.78	10.01 ± 0.73	CG vs HG (0.041∗)
	GIG	5.37 ± 1.64	12.00 ± 1.00	LG vs GIG (0.009∗)
	HG	5.50 ± 0.81	9.64 ± 0.90	LG vs HG (1.00)
				GIG vs HG (<0.001∗)

BDNF level in hippocampus				CG vs LG (0.044∗)
	CG	—	47.68 ± 13.25	CG vs GIG (<0.001∗)
	LG	—	74.46 ± 25.57	CG vs HG (0.032∗)
	GIG	—	112.87 ± 25.18	LG vs GIG (<0.001∗)
	HG	—	76.41 ± 34.68	LG vs HG (0.523)
				GIG vs HG (0.001∗)

BDNF level in striatum				CG vs LG (1.00)
	CG	—	14.16 ± 13.25	CG vs GIG (0.004∗)
	LG	—	18.04 ± 11.61	CG vs HG (1.00)
	GIG	—	27.77 ± 15.57	LG vs GIG (0.044∗)
	HG	—	17.94 ± 10.26	LG vs HG (1.00)
				GIG vs HG (0.030∗)

BDNF level in cortex				CG vs LG (0.980)
	CG	—	11.73 ± 7.18	CG vs GIG (0.001∗)
	LG	—	14.69 ± 3.60	CG vs HG (1.00)
	GIG	—	19.24 ± 4.94	LG vs GIG (0.203)
	HG	—	14.64 ± 6.50	LG vs HG (1.00)
				GIG vs HG (0.194)

Plasma CORT Level				CG vs LG (0.044∗)
	CG	—	347.03 ± 181.02	CG vs GIG (0.009∗)
	LG	—	508.07 ± 161.30	CG vs HG (<0.001∗)
	GIG	—	540.63 ± 117.40	LG vs GIG (1.000)
	HG	—	716.90 ± 156.48	LG vs HG (0.003∗)
				GIG vs HG (0.017∗)

Values: means ± standard deviations; *P* value: significance level of 2-way Repeated Measures ANOVA multiple comparisons with covariate for behavioral scores; significance level of one-way ANOVA for BDNF levels and plasma CORT concentrations.

*Significant differences observed; post hoc test was conducted to specify the differences between groups.
